# Clinical, Behavioral, and Sociodemographic Factors Associated With Self-Reported High Blood Pressure Among Canadian Adults: A Cross-Sectional Analysis of the 2022 Canadian Community Health Survey

**DOI:** 10.7759/cureus.117206

**Published:** 2026-09-30

**Authors:** Oto-obong J Utuk, Adebola A Adeleye, Akinyele Oladimeji, Mariam T Adeyemi, Jabez K Inkoom, Oluwatumininu Akinbohun, Amanda S Boateng, Khadijat Abdulwasiu, Samuel E John, Maharambe E S A D S Ariyaratne

**Affiliations:** 1 General Medicine, Windsor University School of Medicine, Mississauga, CAN; 2 School of Public Health Sciences, University of Waterloo, Waterloo, CAN; 3 Family Medicine, Alberta Health Services, Edmonton, CAN; 4 Internal Medicine, University of Ghana Medical Centre, Accra, GHA; 5 General Medicine, Zaporizhzhia State Medical University, Zaporizhzhia, UKR; 6 Family Medicine, Maternal and Child Health Hospital, Kumasi, GHA; 7 Family and Community Medicine, University of Ilorin Teaching Hospital, Ilorin, NGA; 8 General Practice, Ambrose Alli University, Ekpoma, NGA; 9 Internal Medicine, All Saints Springhill Hospital, Springhill, CAN

**Keywords:** blood pressure, canada, canadian community health survey, cross sectional study, hypertension, risk factors

## Abstract

Background

Hypertension is a common cardiovascular risk factor in Canada. Its distribution may vary according to clinical, behavioural, and sociodemographic characteristics. Although Canadian studies have examined selected hypertension risk factors, contemporary national analyses that assess these domains together among adults remain limited.

Objective

The study aimed to identify clinical, behavioral, and sociodemographic factors associated with self-reported high blood pressure among Canadian adults.

Methods

This cross-sectional study used the 2022 Canadian Community Health Survey public use microdata file. Adults aged 18 years or older with complete data were included. Survey-weighted logistic regression estimated adjusted odds ratios and 95% confidence intervals. Sampling weights and 1,000 bootstrap replicate weights accounted for the complex survey design.

Results

The analytic sample included 52,735 adults, representing 27,062,726 Canadian adults. Self-reported high blood pressure was reported by 5,458,778 (20.2% ) adults. Compared with adults aged 18 to 34 years, adults aged 65 years or older had markedly higher odds of self-reported high blood pressure - adjusted odds ratio: 27.24, 95% confidence interval: 22.00 to 33.73, p < 0.001. Overweight or obesity - adjusted odds ratio: 2.18, 95% confidence interval: 1.99 to 2.39, p < 0.001; diabetes - adjusted odds ratio: 2.49, 95% confidence interval: 2.21 to 2.81, p < 0.001; and fair or poor self-perceived health - adjusted odds ratio: 2.56, 95% confidence interval: 2.28 to 2.87, p < 0.001, were associated with higher odds. Female sex was associated with lower odds than male sex - adjusted odds ratio: 0.88, 95% confidence interval: 0.81 to 0.95, p = 0.001.

Conclusions

Self-reported high blood pressure was associated with age, sex, body mass index category, diabetes, self-perceived health, income, and province group. These findings may assist population health planning and support risk-based blood pressure assessment. Longitudinal studies using measured blood pressure and additional behavioural and clinical variables are needed.

## Introduction

Hypertension is a common chronic condition and an important modifiable risk factor for cardiovascular disease, premature death, and health care use in Canada. In a Canadian primary care study, 57,180 of 250,346 adults (22.8%) had diagnosed hypertension; those with hypertension were 1.34 times as likely to have another chronic condition as those without hypertension [1]. Canadian guidance emphasizes accurate diagnosis, sustained blood pressure control, healthy behaviours, and appropriate pharmacological treatment when indicated [2]. Understanding factors associated with self-reported high blood pressure in the general population can support prevention efforts and help identify groups that may benefit from targeted health promotion and clinical follow-up.

Clinical characteristics are strongly related to hypertension in Canadian adults. Previous Canadian research has identified older age, higher body mass index, diabetes, chronic kidney disease, lower physical activity, and lower fruit and vegetable intake as factors associated with hypertension [3]. National surveillance has also shown that hypertension prevalence, awareness, treatment, and control differ across population groups [4]. These findings support examination of age, body mass index, diabetes, and self-perceived health when studying self-reported high blood pressure in a population survey. Resistance to hypertension remains a clinically important matter, as patients continue to have uncontrolled high blood pressure despite treatment with multiple hypertensive medications; this highlights a need for careful recognition of patients at high risk [5,6]

Moreover, clinical research findings acknowledge the influence of behavioral factors such as smoking, alcohol consumption, diet, and physical inactivity or a generally sedentary lifestyle on hypertension timing and development [7]. More specifically, dietary patterns, especially high consumption of ultra-processed foods, have been linked with risks, development, and progression of hypertension among a Canadian adults’ cohort [8, 9]. However, population modelling research results have shown that reducing sodium consumption can produce positive health results [10]. These behaviors are substantially influenced by the broader food and physical environment. Engaging in simple physical exercises such as daily walking routines has been linked with reducing hypertension prevalence [11]. Dietary changes may also have economic implications: Clair et al. modeled potential healthcare cost savings associated with increased flaxseed consumption among people with hypertension [12].

Differences in hypertension are also shaped by sociodemographic and contextual factors. Research studies suggest that hypertension inequalities exhibit intersections between racial identity, gender, and income factors [13]. However, regional variations of cardiovascular risk factors are linked with socioeconomic status [13, 14]. In the implementation of prevention strategies, population characteristics are necessary due to the variation in cardiovascular risk profiles based on migration and temporal factors [15]. Likewise, financial or income inequalities remain barriers that highly affect treatment outcomes among patients with cardiovascular-related chronic diseases [16]. In addition, occupational effort-reward imbalance has been linked with poor treatment outcomes in the cohort of hypertensive white-collar workers [17]. But positive health outcomes for hypertensive patients may be influenced by language concordance between healthcare professionals and patients [18].

Despite previous studies exploring individual risk factors, treatment, and surveillance of hypertension, contemporary Canadian studies have mainly focused on examining specific cultural, sociodemographic, behavioral, clinical, and environmental factors individually. A review of hypertension surveillance and data quality is warranted because of the varying quality of individually recorded variables, which may affect the accuracy and reliability of the findings of this study [19]. A 2022 Canadian community health survey, for instance, demonstrates the value of contemporary population-level data due to its provision of informed findings from statistical evaluation approaches such as odds ratios, confidence intervals, and prevalence [20]. But these studies have failed to conduct a broader analysis that integrates different domains among Canadian adult populations. Therefore, this study aimed to identify clinical, behavioural, and sociodemographic factors associated with self-reported high blood pressure among Canadian adults using the Canadian Community Health Survey.

## Materials and methods

Study design and data source

This cross-sectional study examined clinical, behavioural, and sociodemographic factors associated with self-reported high blood pressure among Canadian adults. Data were obtained from the 2022 Canadian Community Health Survey (CCHS) public use microdata file [21]. Statistics Canada collected the 2022 survey data from February 7 through December 31, 2022. The Canadian Community Health Survey is a national survey conducted by Statistics Canada that collects information on health status, health behaviours, health care use, and social determinants of health among persons living in private dwellings. The present analysis used the available final sampling weight and 1,000 bootstrap replicate weights supplied with the 2022 dataset.

Study population

The initial 2022 CCHS public-use microdata file contained 67,079 respondents. Participants were eligible for the present analysis if they were aged 18 years or older and had complete information for self-reported high blood pressure and all covariates included in the primary multivariable model. Respondents aged 12-17 years (n = 3,761) were excluded, leaving 63,318 eligible adults. A further 10,583 respondents were excluded because of missing data for the outcome or at least one covariate required for the primary analysis. Complete-case analysis was therefore performed using 52,735 adults, representing a survey-weighted Canadian adult population of 27,062,726. The participant-selection process is shown in Figure 1.

**Figure 1:**
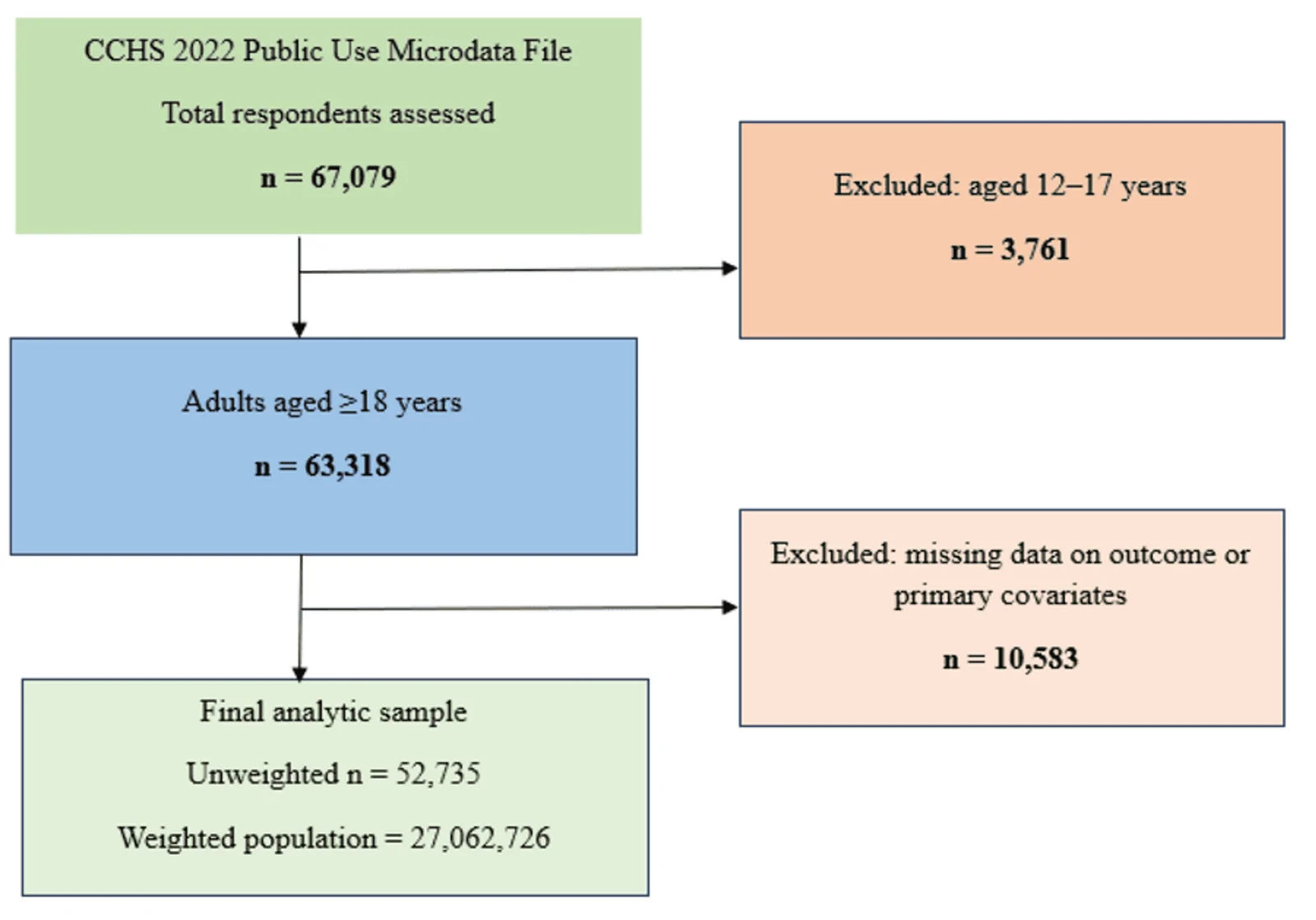
Population selection flow diagram CCHS: Canadian Community Health Survey

Variables and measures

The outcome was self-reported high blood pressure, defined as a respondent’s report that a health professional had diagnosed them with high blood pressure. Respondents reporting no such diagnosis were classified as not having high blood pressure. Clinical factors included body mass index category, self-reported diabetes, and self-perceived health. Diabetes was classified as yes or no. Body mass index (BMI) category was based on the derived BMI classification provided in the 2022 CCHS public-use microdata file and was grouped for analysis as underweight/normal weight and overweight/obese [21]. Self-perceived health was categorized as excellent or very good, good, and fair or poor. Behavioural factors included smoking status and alcohol consumption. Smoking status was categorized as current smoker, former smoker, or never smoker. Alcohol consumption was categorized as regular drinker, occasional drinker, or non-drinker. Sociodemographic factors included sex, age group, educational attainment, household income quintile, and province or territory group. Age was categorized as 18 to 34 years, 35 to 49 years, 50 to 64 years, and 65 years or older. Educational attainment was categorized as less than secondary education, secondary education, and post-secondary education. Household income was categorized into five quintiles. Province or territory was initially grouped into seven categories: Atlantic Canada, Quebec, Ontario, Prairies, Alberta, British Columbia, and the territories. Respondents from the territories were not represented in the final complete-case analytic sample after exclusion of respondents with missing data for the outcome or primary covariates. Consequently, the final analyses included six geographic categories: Atlantic Canada, Quebec, Ontario, Prairies, Alberta, and British Columbia. Cardiovascular disease was considered during initial variable selection but was not included in the primary all-adult analysis because its derived measure was structurally unavailable for many respondents aged 18 to 34 years. Including this variable in complete-case analysis removed the youngest age group from the analytic sample.

Missing data

Missing data were assessed before complete-case analysis. Missingness was 3.33% for education, 5.16% for body mass index category, 0.39% for alcohol consumption, 4.27% for household income quintile, 0.78% for self-reported high blood pressure, 1.30% for diabetes, 0.08% for self-perceived health, and 3.26% for smoking status. Age group, sex, and province or territory group had 0.00% missing data. The initially considered cardiovascular disease variable had 18.43% structural nonavailability after recoding, concentrated among adults aged 18 to 34 years, and was therefore excluded from the primary model. Complete-case analysis was applied to self-reported high blood pressure and the primary covariates. This resulted in exclusion of 10,583 of 63,318 eligible adults, or 16.71%, and retained 52,735 adults for the primary analysis. Following complete-case restriction, no respondents from the territories remained in the analytic sample; therefore, the territories were not represented as a separate category in the final regression analysis.

Statistical analysis

All analyses accounted for the complex survey design of the 2022 CCHS. The final survey sampling weight was applied to obtain population-representative estimates, and the 1,000 bootstrap replicate weights supplied with the dataset were used for variance estimation. Descriptive characteristics were summarized by self-reported high blood pressure status using weighted frequencies and column percentages for sex, age group, education, household income quintile, province group, BMI category, smoking status, alcohol consumption, diabetes, and self-perceived health. Formal group comparisons were not conducted because bootstrap replicate weighting in descriptive cross-tabulations may not provide stable inference for conventional hypothesis testing procedures. Survey-weighted multivariable logistic regression was used to estimate adjusted odds ratios and 95% confidence intervals for factors associated with self-reported high blood pressure. The model included sex, age group, education, household income quintile, province or territory group, body mass index category, diabetes, smoking status, alcohol consumption, and self-perceived health. Multicollinearity was assessed using variance inflation factors (VIF). VIF values ranged from 1.05 to 3.37, with a mean VIF of 1.94, indicating no important multicollinearity among the covariates. Analyses were conducted using Stata (StataCorp LLC, College Station, TX, USA; 2023) [22].

Ethical considerations

This study involved secondary analysis of anonymized public-use microdata from the 2022 Canadian Community Health Survey. The dataset contained no direct personal identifiers, and there was no contact with participants. The analysis used data that were legally accessible in de-identified form and did not involve information for which participants had a reasonable expectation of privacy.

## Results

Table 1 presents the weighted characteristics of Canadian adults by self-reported high blood pressure status. The analytic sample included 52,735 respondents and represented 27,062,726 Canadian adults after application of survey weights.

**Table 1:** Weighted clinical, behavioural, and sociodemographic characteristics of Canadian adults by self-reported high blood pressure status Values are weighted counts and column percentages, reported as n (%). BMI means body mass index. Estimates accounted for the final sampling weights and 1,000 bootstrap replicate weights from the 2022 Canadian Community Health Survey. Formal group comparisons were not conducted because bootstrap replicate weighting in descriptive cross-tabulations may not provide stable inference for conventional hypothesis testing procedures. The symbol (–) indicates intentionally left blank. All analyses were performed using Stata version 18 (StataCorp LLC, College Station, TX, USA; 2023) [22].

Variable	No high blood pressure, n (%) N = 21,603,948	Self-reported high blood pressure, n (%) N = 5,458,778
Sex, n (%)	–	–
Male	10,777,309 (49.89%)	2,846,585 (52.15%)
Female	10,826,639 (50.11%)	2,612,193 (47.85%)
Age group, n (%)	–	–
18-34 years	7,194,155 (33.30%)	162,380 (2.97%)
35-49 years	6,003,858 (27.79%)	661,000 (12.11%)
50-64 years	4,963,720 (22.98%)	1,820,589 (33.35%)
65+ years	3,442,215 (15.93%)	2,814,809 (51.57%)
Education level, n (%)	–	–
Less than secondary	718,263 (3.32%)	469,058 (8.59%)
Secondary education	2,926,027 (13.54%)	1,155,395 (21.17%)
Post-secondary education	17,959,658 (83.14%)	3,834,325 (70.24%)
Income quintile, n (%)	–	–
Lowest quintile	3,896,548 (18.04%)	1,286,145 (23.56%)
Second quintile	4,129,449 (19.11%)	1,160,921 (21.27%)
Middle quintile	4,399,863 (20.37%)	1,072,981 (19.66%)
Fourth quintile	4,546,777 (21.05%)	995,295 (18.23%)
Highest quintile	4,631,311 (21.43%)	943,436 (17.28%)
Province group, n (%)	–	–
Atlantic Canada	1,294,126 (5.99%)	426,228 (7.81%)
Quebec	4,991,097 (23.10%)	1,204,834 (22.07%)
Ontario	8,493,149 (39.31%)	2,193,405 (40.18%)
Prairies	1,242,012 (5.75%)	333,884 (6.12%)
Alberta	2,538,227 (11.75%)	582,790 (10.68%)
British Columbia	3,045,337 (14.10%)	717,637 (13.14%)
BMI category, n (%)	–	–
Underweight/normal weight	8,463,759 (39.18%)	1,104,114 (20.23%)
Overweight/obese	13,140,189 (60.82%)	4,354,664 (79.77%)
Smoking status, n (%)	–	–
Current smoker	2,392,631 (11.07%)	585,166 (10.72%)
Former smoker	4,464,395 (20.67%)	1,810,646 (33.17%)
Never smoker	14,746,922 (68.26%)	3,062,966 (56.11%)
Alcohol consumption, n (%)	–	–
Regular drinker	12,874,271 (59.59%)	2,911,037 (53.33%)
Occasional drinker	3,957,018 (18.32%)	1,066,401 (19.54%)
Non-drinker	4,772,659 (22.09%)	1,481,340 (27.13%)
Diabetes, n (%)	–	–
No diabetes	20,679,870 (95.72%)	4,272,538 (78.27%)
Diabetes	924,078 (4.28%)	1,186,240 (21.73%)
Self-perceived health, n (%)	–	–
Excellent/very good	12,966,292 (60.02%)	1,874,122 (34.33%)
Good	6,280,535 (29.07%)	2,226,366 (40.79%)
Fair/poor	2,357,121 (10.91%)	1,358,290 (24.88%)

Self-reported high blood pressure was reported by 5,458,778 Canadian adults, representing approximately one-fifth of the weighted analytic population. Adults with self-reported high blood pressure were more commonly aged 65 years or older, overweight or obese, living with diabetes, and reporting good, fair, or poor health than adults without high blood pressure. Adults aged 18 to 34 years accounted for a small proportion of those reporting high blood pressure. The high blood pressure group also had a greater proportion of former smokers, participants in the lowest income quintile, and participants with less than secondary education. The sex distribution and provincial distribution were similar across the two groups, although the high blood pressure group included a slightly greater proportion of males and residents of Atlantic Canada.

Table 2 presents the adjusted associations between clinical, behavioural, and sociodemographic factors and self-reported high blood pressure.

**Table 2:** Adjusted associations of clinical, behavioural, and sociodemographic factors with self-reported high blood pressure among Canadian adults Adjusted odds ratios and 95% confidence intervals were estimated using survey-weighted multivariable logistic regression. The model included sex, age group, education, income quintile, province group, BMI category, diabetes, smoking status, alcohol consumption, and self-perceived health. OR means odds ratio. CI means confidence interval. Reference groups were male sex, age 18 to 34 years, less than secondary education, lowest income quintile, Atlantic Canada, underweight or normal weight, no diabetes, current smoking, regular drinking, and excellent or very good self-perceived health. An asterisk (*) indicates statistical significance at p < 0.05. The symbol (–) indicates intentionally left blank. All analyses were performed using Stata version 18 (StataCorp LLC, College Station, TX, USA; 2023) [22].

Variable	Adjusted OR	95% CI	p value
Sex	–	–	–
Female vs. male	0.88	0.81 to 0.95	0.001*
Age group	–	–	–
35-49 years vs. 18–34 years	4.22	3.36 to 5.30	<0.001*
50-64 years vs. 18–34 years	12.98	10.45 to 16.11	<0.001*
65+ years vs. 18–34 years	27.24	22.00 to 33.73	<0.001*
Education level	–	–	–
Secondary education vs. less than secondary	1.01	0.87 to 1.17	0.936
Post-secondary education vs. less than secondary	0.90	0.78 to 1.03	0.111
Income quintile	–	–	–
Second quintile vs. lowest quintile	0.93	0.82 to 1.05	0.235
Middle quintile vs. lowest quintile	0.88	0.77 to 1.00	0.048*
Fourth quintile vs. lowest quintile	0.92	0.82 to 1.04	0.174
Highest quintile vs. lowest quintile	0.83	0.73 to 0.95	0.005*
Province group	–	–	–
Quebec vs. Atlantic Canada	0.84	0.74 to 0.95	0.004*
Ontario vs. Atlantic Canada	0.99	0.89 to 1.11	0.910
Prairies vs. Atlantic Canada	1.04	0.90 to 1.21	0.589
Alberta vs. Atlantic Canada	0.97	0.85 to 1.10	0.617
British Columbia vs. Atlantic Canada	0.87	0.76 to 1.01	0.066
BMI category	–	–	–
Overweight/obese vs. underweight/normal weight	2.18	1.99 to 2.39	<0.001*
Diabetes	–	–	–
Diabetes vs. no diabetes	2.49	2.21 to 2.81	<0.001
Smoking status	–	–	–
Former smoker vs. current smoker	1.07	0.93 to 1.23	0.357
Never smoker vs. current smoker	1.08	0.94 to 1.24	0.282
Alcohol consumption	–	–	–
Occasional drinker vs. regular drinker	1.07	0.96 to 1.19	0.198
Non-drinker vs. regular drinker	0.96	0.86 to 1.06	0.375
Self-perceived health	–	–	–
Good vs. excellent/very good	1.96	1.80 to 2.13	<0.001*
Fair/poor vs. excellent/very good	2.56	2.28 to 2.87	<0.001*

As shown in Table 2, after multivariable adjustment, female adults had lower odds of self-reported high blood pressure than male adults (adjusted odds ratio (aOR) 0.88, 95% CI 0.81-0.95, p = 0.001). Compared with adults aged 18-34 years, the adjusted odds increased progressively among those aged 35-49 years (aOR 4.22, 95% CI 3.36-5.30, p < 0.001), 50-64 years (aOR 12.98, 95% CI 10.45-16.11, p < 0.001), and ≥65 years (aOR 27.24, 95% CI 22.00-33.73, p < 0.001). Overweight or obesity (aOR 2.18, 95% CI 1.99-2.39, p < 0.001), diabetes (aOR 2.49, 95% CI 2.21-2.81, p < 0.001), good self-perceived health (aOR 1.96, 95% CI 1.80-2.13, p < 0.001), and fair or poor self-perceived health (aOR 2.56, 95% CI 2.28-2.87, p < 0.001) were also associated with higher odds. Adults in the middle-income (aOR 0.88, 95% CI 0.77-1.00, p = 0.048) and highest-income (aOR 0.83, 95% CI 0.73-0.95, p = 0.005) quintiles had lower odds than those in the lowest quintile, while adults residing in Quebec had lower odds than those in Atlantic Canada (aOR 0.84, 95% CI 0.74-0.95, p = 0.004). Education, smoking status, alcohol consumption, and the remaining province groups were not statistically associated with self-reported high blood pressure after adjustment.

Figure 2 illustrates the weighted age-specific predicted probability of self-reported high blood pressure among Canadian adults.

**Figure 2:**
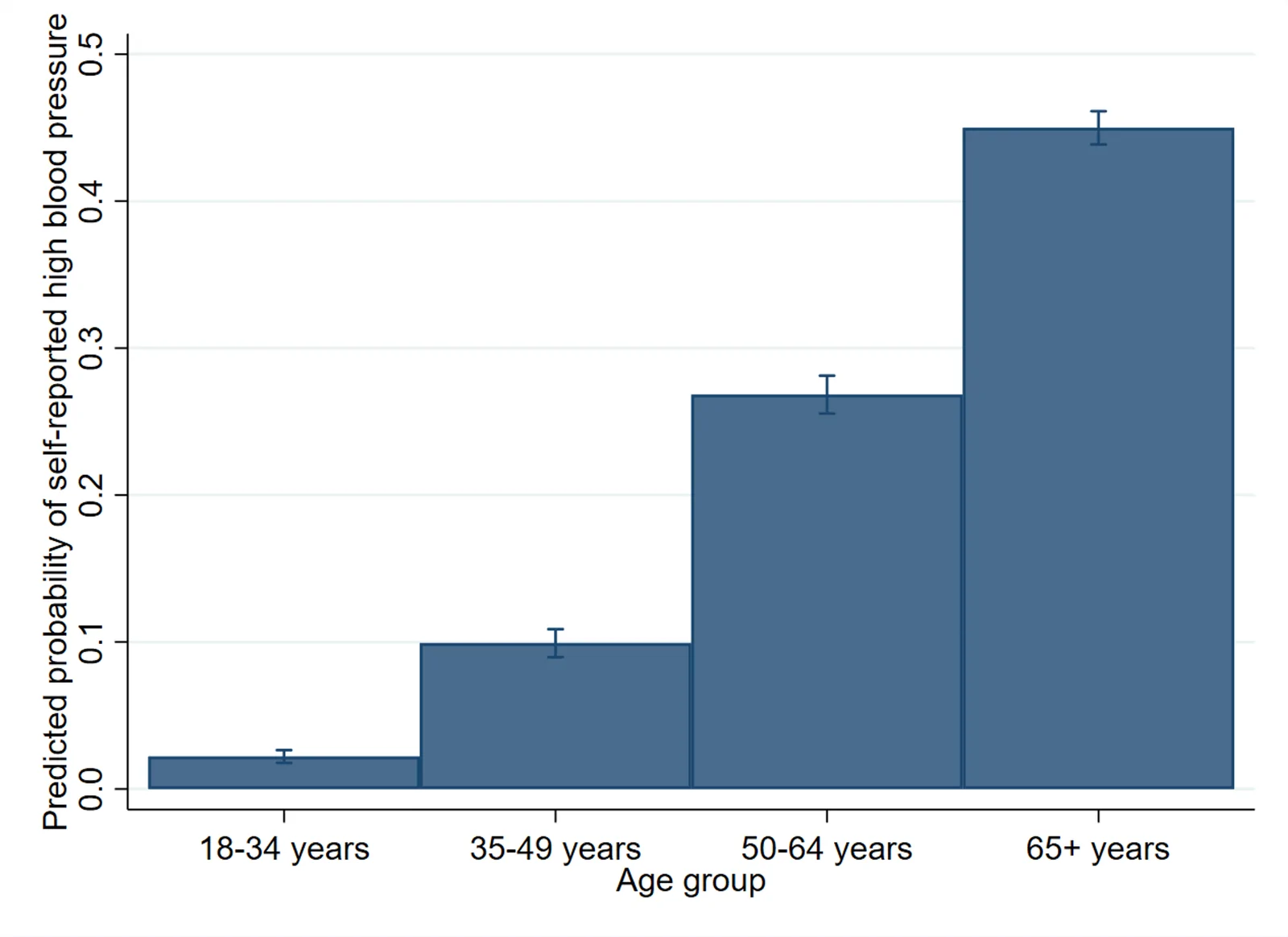
Predicted probability of self-reported high blood pressure by age group among Canadian adults Bars show survey-weighted predicted probabilities from a logistic regression model containing age group. Error bars show 95% confidence intervals estimated using 1,000 bootstrap replicate weights from the 2022 Canadian Community Health Survey (CCHS). The outcome was self-reported high blood pressure. The predicted probabilities and 95% confidence intervals were estimated using Stata Statistical Software, Release 18 (StataCorp LLC, College Station, TX, USA; 2023) [22], using the CCHS final sampling weight and 1,000 bootstrap replicate weights.

The predicted probability of self-reported high blood pressure increased steadily with age. It was lowest among adults aged 18 to 34 years and highest among adults aged 65 years or older. The rise was most pronounced between the 35-49-year and 50-64-year groups, with a further increase among adults aged 65 years or older.

## Discussion

In this study, self-reported high blood pressure was present in approximately one-fifth of the weighted adult population. Older age, overweight or obesity, diabetes, and poorer self-perceived health were independently associated with higher odds of self-reported high blood pressure. Female sex, residence in Quebec, and membership in the middle- or highest-income quintile were associated with lower odds. The strongest association was observed for age 65 years or older compared with age 18 to 34 years. These findings are consistent with Canadian evidence identifying age, body mass index, diabetes, and other chronic conditions as important correlates of hypertension [3,5,14]. Education, smoking status, alcohol consumption, and most provincial groups were not independently associated with the outcome in the adjusted model.

The 2025 Canadian hypertension guidance recommends accurate blood pressure assessment, evaluation of cardiovascular and kidney risk, lifestyle management, and individualized pharmacological treatment when indicated [2]. The present findings are relevant to this approach because the groups with higher adjusted odds of self-reported high blood pressure included older adults, adults with overweight or obesity, and adults with diabetes. It also emphasizes management of excess weight, diabetes, chronic kidney disease, and resistant hypertension. Although this study did not measure blood pressure directly or assess treatment, it identifies population groups in which accurate measurement, clinical assessment, and continued follow-up may be particularly important. Canadian guidance similarly stresses risk assessment and management of behavioural and clinical factors in hypertension care [2,5].

The odds of self-reported high blood pressure increased markedly with age. This pattern is consistent with age-related increases in arterial stiffness and changes in vascular function. Canadian data have shown a higher burden of hypertension among older adults, as well as greater treatment and comorbidity among older groups [3,5]. The association between overweight or obesity and high blood pressure may reflect pathways involving increased vascular resistance, insulin resistance, and activation of hormonal systems that regulate blood pressure. The association with diabetes is also clinically plausible because diabetes and hypertension frequently occur together and share metabolic and vascular risk factors [3,5]. Canadian research among Indigenous adults using the 2022 Canadian Community Health Survey also reported a positive association between body mass index and hypertension [20]. These patterns do not establish causation but support integrated attention to blood pressure, weight, and diabetes in clinical and public health settings.

Income and geographic differences remained after adjustment. Adults in the middle- and highest-income quintiles had lower odds of self-reported high blood pressure than those in the lowest quintile, and adults living in Quebec had lower odds than those living in Atlantic Canada. These findings are consistent with Canadian reports of socioeconomic and regional variation in hypertension and cardiovascular risk factors [13,14]. Financial barriers may affect access to medication, healthy food, and chronic disease care among people with cardiovascular-related conditions [16]. The lower adjusted odds observed in Quebec may reflect differences in population composition, health behaviours, health care use, or diagnosis patterns. The survey data cannot determine which of these factors accounts for the observed geographic association.

From a public-health perspective, these findings support continued efforts to improve hypertension awareness and blood pressure assessment among Canadian adults, particularly in groups with a greater burden of self-reported high blood pressure. Risk-factor strategies addressing excess body weight, diabetes, and socioeconomic disadvantage may complement population-wide hypertension prevention and awareness initiatives. The observed age and regional differences also suggest that prevention and screening approaches may benefit from consideration of population characteristics and local context.

Smoking status and alcohol consumption were not statistically associated with self-reported high blood pressure after adjustment. This differs from literature describing smoking, alcohol use, diet, and physical activity as behavioural factors related to chronic disease and hypertension risk [7]. The adjusted findings may reflect differences in age, body weight, diabetes, health status, or patterns of self-reporting across these groups. The study did not include dietary sodium, fruit and vegetable intake, physical activity, or detailed measures of alcohol quantity. Canadian studies have linked ultra-processed foods, sodium intake, neighbourhood food environments, and walkability with hypertension-related outcomes [8,9,10,11]. These factors remain relevant for prevention, even though they were not examined in the present model.

Strengths and limitations of the study

This study had a large national sample and used survey weights and bootstrap variance estimation. Several limitations should be considered. The Canadian Community Health Survey samples people living in private dwellings, which limits representation of some populations. High blood pressure was defined by a respondent’s report of a diagnosis from a health professional; no measured blood pressure threshold was applied. This outcome may therefore be affected by differences in diagnosis awareness and access to care, as well as reporting error. Diabetes, smoking, alcohol use, body mass index category, and self-perceived health were also self-reported. Complete-case analysis excluded 16.71% of eligible adults because of missing data. If excluded adults differed from those retained, selection bias may have affected the estimates; its direction and magnitude cannot be determined from this analysis. Cardiovascular disease was omitted because the variable was structurally unavailable for younger adults. Measured blood pressure, medication use, diet, sodium intake, physical activity, chronic kidney disease, race, migration status, and healthcare access were not included. The cross-sectional design cannot establish temporal order or causation. Future longitudinal studies should include measured blood pressure and more detailed clinical, behavioural, and social data.

## Conclusions

This study identified clinical, behavioural, and sociodemographic factors associated with self-reported, previously diagnosed high blood pressure among Canadian adults. Older age, male sex, overweight or obesity, diabetes, and poorer self-perceived health were associated with higher odds; associations also differed by income and region. Because the study was cross-sectional and blood pressure was not measured, these findings do not establish causation or identify undiagnosed hypertension. They may help guide blood pressure assessment, but the results should be interpreted in light of self-reporting and complete-case exclusion. Future longitudinal studies should include measured blood pressure and information on medication use, diet, physical activity, and healthcare access.
